# Mandibular buccal shelf morphology among Indian adults using CBCT across class I, class II and class III malocclusions

**DOI:** 10.6026/973206300214372

**Published:** 2025-12-15

**Authors:** Sushil Bhagwan Mahajan, Vinay V. Bedre, Trilok Shrivastav, Zeeshan Mubashir, Swati Swati, Manawar Ahmad Mansoor, Hina Naim Abdul

**Affiliations:** 1 Department of Orthodontics and Dentofacial Orthopaedics, People's University Bhopal, Madhya Pradesh, India; 2 Department of Orthodontics and Dentofacial Orthopaedics, Al Jouf Specialised Dental Center, Ministry of Health, Sakaka, Aljouf Province, Kingdom of Saudi Arabia; 3 Department of Oral Pathology, Swami Devi Dyal Hospital and Dental College, Panchkula, Haryana, India; 4 Department of Prosthetic Dental Sciences, College of Dentistry, Jazan University, Kingdom of Saudi Arabia

**Keywords:** Cone-beam computed tomography, mandible, orthodontic anchorage procedures, bone density, malocclusion

## Abstract

The mandibular buccal shelf (MBS) is recognized as a stable site for miniscrew insertion due to its dense cortical bone, although its morphology varies across malocclusion types. Therefore, it is of interest to compare buccal shelf width and cortical bone thickness among adults with Class I, Class II and Class III skeletal patterns using cone-beam computed tomography (CBCT). A retrospective analysis of 80 CBCT scans was performed, with measurements taken at standardized apical levels and statistically compared using one-way ANOVA. Results showed that Class III subjects exhibited significantly greater buccal shelf width and cortical thickness than Class I and Class II groups, indicating more favorable bone morphology for orthodontic anchorage. These findings suggest that MBS characteristics are most advantageous in Class III patients, highlighting the importance of individualized CBCT-based assessment for miniscrew planning.

## Background:

Temporary anchorage devices (TADs) have greatly advanced orthodontic biomechanics, allowing absolute anchorage with minimal reliance on patient compliance [1]. Among the available sites, the mandibular buccal shelf (MBS) has become popular because of its accessibility, distance from dental roots and dense cortical bone [2]. The stability of miniscrews depends primarily on adequate bone quality and cortical thickness [3]. Differences in mandibular morphology across skeletal malocclusions may alter the suitability of the buccal shelf for miniscrew insertion [4]. Class III patients typically exhibit greater mandibular width and cortical development, while Class II patients may show reduced alveolar support [5]. Cone-beam computed tomography (CBCT) offers precise three-dimensional evaluation of cortical bone thickness and width, improving preoperative planning and reducing risks associated with mini screw placement [6]. Earlier studies highlight that optimal miniscrew placement zones vary by skeletal class, growth pattern and patient age, underscoring the need for individualized evaluation [7]. Therefore, it is of interest to report and compare the morphology of the mandibular buccal shelf among Class I, Class II and Class III malocclusion groups in an adult population using CBCT.

## Materials and Methods:

A retrospective cross-sectional study was conducted to evaluate CBCT images for buccal shelf morphology. Eighty adult patients (26 Class I, 27 Class II and 27 Class III) aged between 18-45 years were included. Classification was based on Angle's criteria. The included subjects were those that had Borderline extraction/surgical Class III cases; Adults requiring anchorage in regular or mutilated malocclusion cases and Patients undergoing midline correction. The excluded patients were those with Systemic bone disease, previous orthognathic surgery, Severe periodontal disease; Conditions affecting bone metabolism; Craniofacial anomalies or bone pathology and Contraindications to CBCT. CBCT scans were obtained under standardized conditions (90 kVp, 10 mA, voxel size 0.3 mm). Measurements were taken at the mandibular first molar region. Buccal shelf width (mm) that is the Perpendicular distance from buccal cortical plate to nearest root surface at mandibular first/second molars and Cortical bone thickness (mm) that is Measured 4 mm, 6 mm and 8 mm apical to alveolar crest were measured. Descriptive statistics (mean ± SD) were computed. Intergroup comparisons were performed using one-way ANOVA and Tukey's post-hoc test, with significance set at p < 0.05.

## Results:

Analysis of buccal shelf width revealed clear intergroup differences. Class III patients presented the highest mean width (7.90 ± 1.20 mm), followed by Class I (7.25 ± 1.12 mm), while Class II demonstrated the lowest values (6.85 ± 1.05 mm). The difference reached statistical significance (p = 0.04). These results suggest that the buccal shelf in Class III provides a broader cortical platform for mini screw insertion compared to the narrower shelf observed in Class II Table 1 (see PDF). Cortical bone thickness measurements at 4 mm, 6 mm and 8 mm apical to the alveolar crest showed consistent trends across all skeletal classes. Thickness increased progressively with depth, reflecting improved cortical support in more apical regions. Class III patients displayed the thickest cortical bone at all levels: 2.10 ± 0.45 mm at 4 mm, 2.35 ± 0.50 mm at 6 mm and 2.60 ± 0.48 mm at 8 mm. Class I patients showed intermediate values, while Class II consistently exhibited the lowest measurements (1.70 ± 0.35 mm at 4 mm, 1.95 ± 0.42 mm at 6 mm and 2.15 ± 0.40 mm at 8 mm). Intergroup differences were statistically significant at all levels (p < 0.05). The occlusal CBCT view further illustrates the distribution of potential insertion zones in the mandibular buccal shelf (Table 2 - see PDF).

## Discussion:

This study assessed MBS dimensions among Class I, II and III skeletal malocclusions using CBCT. Results showed Class III patients possessed the most favorable osseous conditions for mini screw placement, while Class II patients presented the least favorable parameters. The significantly greater buccal shelf width in Class III patients can be attributed to mandibular prognathism and wider transverse mandibular dimensions [8]. These characteristics enhance cortical plate development and improve stability for mini screws. Conversely, the reduced shelf width and thinner cortical bone in Class II patients correlate with mandibular retrognathism, resulting in a limited anchorage zone [9]. The finding that cortical bone thickness increases apically is consistent with prior studies, which identified improved cortical support at deeper insertion points [10]. This supports recommendations for mini screw placement 6-8 mm apical to the alveolar crest to maximize stability [11]. Comparison with other CBCT studies indicates that variations in skeletal morphology strongly influence mini screw stability. Class III patients generally exhibit increased buccal bone dimensions compared to Class II, reaffirming that skeletal class is a critical factor in TAD planning [12]. The present study also highlights the importance of considering site selection in borderline extraction and surgical cases. Reduced bone availability in Class II patients necessitates careful planning, possibly requiring alternative insertion sites or longer mini screws to ensure primary stability [13]. Clinically, these findings underscore the utility of CBCT imaging for individualized assessment prior to TAD insertion. Accurate evaluation of shelf width and cortical thickness ensures safer placement and minimizes risks such as screw loosening or root contact [14, 15]. Limitations of this study include its retrospective design and relatively small sample size within each malocclusion category. Further multicenter trials with larger and more diverse populations are needed to confirm these results and establish standardized guidelines.

## Conclusion:

Mandibular buccal shelf morphology varies significantly across skeletal malocclusions. Class III patient's exhibit the most favorable dimensions for mini screw insertion, while Class II patients have reduced cortical thickness and shelf width. Cortical bone thickness increases apically, supporting deeper insertion for improved anchorage. CBCT is essential for accurate diagnosis and individualized orthodontic planning.
